# Supplementary material for: Strongly deleterious mutations influence reproductive output and longevity in an endangered population
Source: Nat Commun. 2024 Sep 27;15:8378. doi: 10.1038/s41467-024-52741-4 (PMC11436772; doi:10.1038/s41467-024-52741-4)
Supplement: Supplementary file 1 — Supplementary Information [file 41467_2024_52741_MOESM1_ESM.pdf]

Supplementary material

Strongly deleterious mutations influence reproductive output and longevity in an endangered population

Malin Hasselgren, Nicolas Dussex, Johanna von Seth, Anders Angerbjörn, Love Dalén & Karin Norén

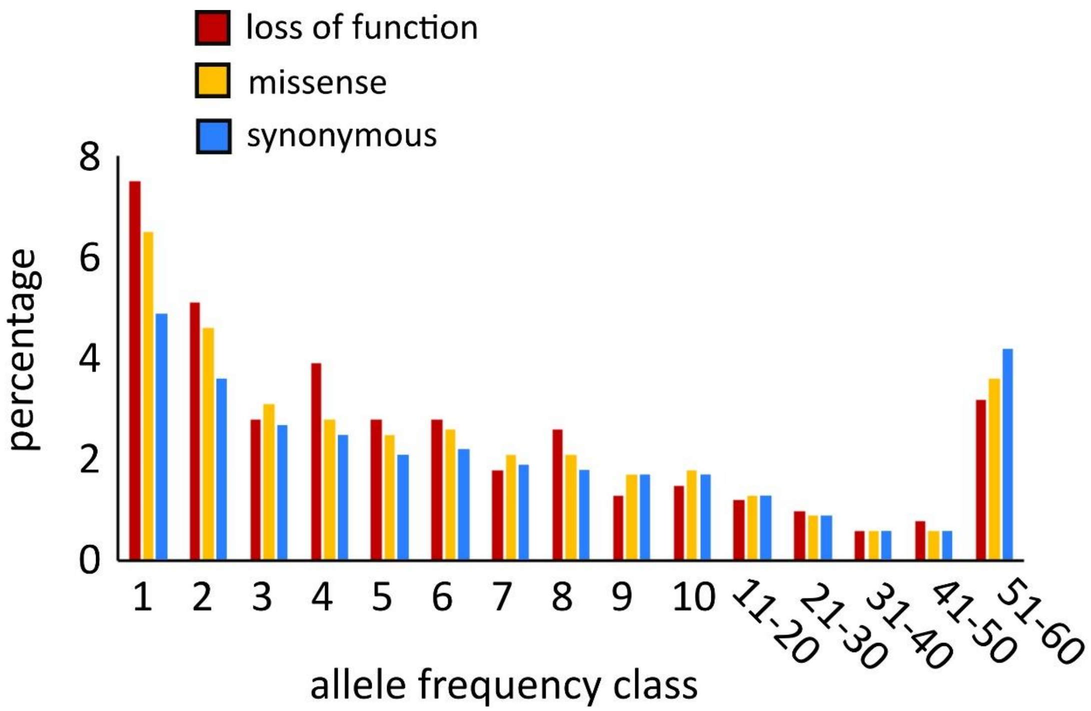

Figure S1: Site frequency spectrum for loss of function, missense and synonymous alleles in a Swedish arctic fox population. Allele frequency classes >10 are shown as means per interval. n = 1300 LoF sites, 44 433 missense sites, 86 508 synonymous sites. Source data are provided as a source data file.

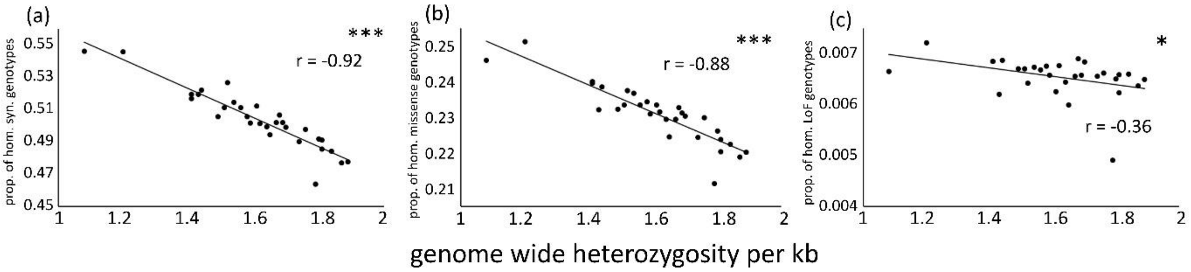

Figure S2: Correlations between genome wide heterozygosity and the proportion of homozygous synonymous (a; p = 1.20 x 10^{-15}), missense (b; p = 4.9 x 10^{-13}) and loss of function (c; p = 0.029) genotypes in an arctic fox population. * marks statistically significant results (***p < 0.001, *p < 0.05; two-sided correlation tests; n = 30 individuals). P-values were not adjusted for multiple testing. Source data are provided as a source data file.

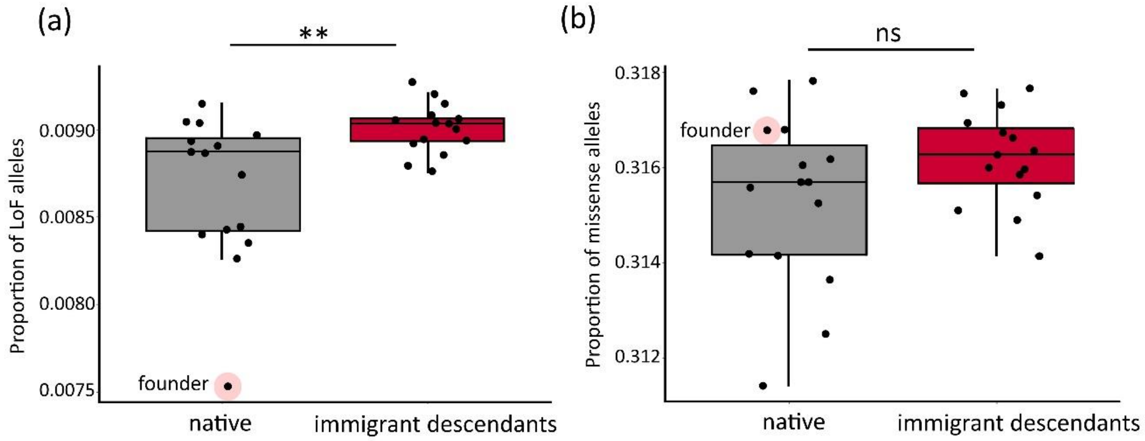

Figure S3: Proportion of loss of function (LoF; a; p = 0.009) and missense (b; p = 0.217) mutations in native individuals and immigrant descendants in a Swedish arctic fox population. * marks statistically significant results (*p < 0.05, ns = non-significant; two-sided Mann-Whitney U-tests). P-values were not adjusted for multiple testing. n = 30 (15 native and 15 hybrid individuals). The red circle marks a female founder ear tagged in 2001. Source data are provided as a source data file.

Table S1: Ancestry, sex, year of birth and depth of coverage of the 30 whole genome sequenced arctic foxes

ID	Ancestry	Sex	Year of birth	Coverage
01003	native	f	unknown	24.90
04043	native	f	2004	26.28
05043	native	m	unknown	25.00
06002	native	m	2006	23.56
08051	native	f	2008	26.13
10026	native	m	2010	25.49
10033	native	f	2010	25.21
10056	native	f	2010	23.63
10060	native	m	2010	26.04
10068	native	f	2010	38.79
10072	native	f	2010	23.02
10075	native	m	2010	23.01
10079	immigrant F1	f	2010	28.69
11102	immigrant F1	m	2011	25.83
11044	immigrant F2	f	2011	28.35
11087	immigrant F1	f	2011	36.69
11090	immigrant F1	m	2011	32.38
13011	immigrant F2	m	2013	18.66
13028	immigrant F2	m	2013	23.93
13036	immigrant F1	m	2011	26.47
13051	native	f	2013	26.32
14005	immigrant F2	m	2014	24.42
14039	immigrant F2	m	2014	29.91
14056	native	f	2014	26.16
15004	immigrant F2	m	2015	21.08
15013	native	f	2015	28.29
15060	immigrant F3	f	2015	27.33
15075	immigrant F3	m	2015	28.45
18005	immigrant F3	m	2018	23.76
18049	immigrant F3	m	2018	31.19

Table S2: Results from two-sided linear model (square.root.LRS ~ LoF.hom + phase + sex, data = mutations_fitness) where bold values indicate significant results. Asterisks show level of significance as P<0.001 ***, P<0.01 ** and P<0.05 *.

**Coefficents**	Estimate	Std. Error	t-value	p-value
Intercept	23.9995	9.6988	2.474	**0.0268***
LoF.hom	-3326.99	1482.3934	-2.244	**0.0415***
phase: low	-0.4035	0.6291	-0.641	0.5317
sex: male	0.4946	0.6169	0.802	0.4361

Table S3: Results from two-sided linear model (log.age ~ LoF.hom + phase + sex, data = mutations_fitness) where bold values indicate significant results. Asterisks show level of significance as P<0.001 ***, P<0.01 ** and P<0.05 *.

**Coefficents**	Estimate	Std. Error	t-value	p-value
Intercept	1.093e+01	3.204e+00	3.411	**0.00332****
LoF.hom	-1.515e+03	4.897e+02	-3.094	**0.00659****
phase: low	-1.643e-01	2.414e-01	-0.680	0.50546
sex: male	2.556e+02	2.359e-01	0.108	0.91496

Table S4: Results from two-sided linear model (litter.size ~ LoF.hom + phase + sex, data = mutations_fitness).

**Coefficents**	Estimate	Std. Error	t-value	p-value
Intercept	30.9848	20.4429	1.516	0.152
LoF.hom	-4184.0200	3124.5480	-1.339	0.202
phase: low	-0.4440	1.3260	-0.335	0.743
sex: male	0.7495	1.3003	0.576	0.574

Table S5: Results from two-sided generalized linear model (survival ~ LoF.hom + phase + sex, family = binomial, data = mutations_fitness).

**Coefficents**	Estimate	Std. Error	z-value	p-value
Intercept	22.1650	11.8147	1.876	0.0606
LoF.hom	-3300.2431	1770.1475	-1.864	0.0623
phase: low	0.1948	0.7664	0.254	0.7994
sex: male	-0.1984	0.7504	-0.264	0.7915

Table S6: Results from two-sided linear model (square.root.LRS ~ missense.hom + phase + sex, data = mutations_fitness).

**Coefficents**	Estimate	Std. Error	t-value	p-value
Intercept	17.2206	14.4907	1.188	0.254
missense.hom	-65.2220	63.1147	-1.033	0.319
phase: low	-0.5092	0.7081	-0.719	0.484
sex: male	0.3064	0.7085	0.432	0.672

Table S7: Results from two-sided linear model (log.age ~ missense.hom + phase + sex, data = mutations_fitness).

**Coefficents**	Estimate	Std. Error	t-value	p-value
Intercept	11.69176	5.97497	1.957	0.0670
missense.hom	-46.45664	26.02396	-1.785	0.0921
phase: low	-0.07708	0.27364	-0.282	0.7816
sex: male	-0.11655	0.27698	-0.421	0.6792

Table S8: Results from two-sided linear model (litter.size ~ missense.hom + phase + sex, data = mutations_fitness).

**Coefficents**	Estimate	Std. Error	t-value	p-value
Intercept	19.5450	28.5458	0.685	0.505
missense.hom	-69.3205	124.3316	-0.558	0.586
phase: low	-0.5675	1.3949	-0.407	0.690
sex: male	0.5422	1.3956	0.389	0.703

Table S9: Results from two-sided generalized linear model (survival ~ missense.hom + phase + sex, family = binomial, data = mutations_fitness) where bold values indicate significant results. Asterisks show level of significance as P<0.001 ***, P<0.01 ** and P<0.05 *.

**Coefficents**	Estimate	Std. Error	z-value	p-value
Intercept	47.0898	19.4790	2.417	**0.0156***
missense.hom	-200.2072	83.0370	-2.411	**0.0159***
phase: low	-0.5285	0.9014	-0.586	0.5577
sex: male	-0.5411	0.8420	-0.643	0.5205

Table S10: Results from two-sided linear model (square.root.LRS ~ synonymous.hom + phase + sex, data = mutations_fitness).

**Coefficents**	Estimate	Std. Error	t-value	p-value
Intercept	13.7603	15.1002	0.911	0.378
synonymous.hom	-22.9733	30.1343	-0.762	0.458
phase: low	-0.5381	0.7253	-0.742	0.470
sex: male	0.3364	0.7224	0.466	0.649

Table S11: Results from two-sided linear model (log.age ~ synonymous.hom + phase + sex, data = mutations_fitness).

**Coefficents**	Estimate	Std. Error	t-value	p-value
Intercept	7.34262	6.58018	1.116	0.280
synonymous.hom	-12.59683	13.12746	-0.960	0.351
phase: low	-0.04885	0.29117	-0.168	0.869
sex: male	-0.08081	0.29657	-0.272	0.789

Table S12: Results from two-sided linear model (litter.size ~ synonymous.hom + phase + sex, data = mutations_fitness).

**Coefficents**	Estimate	Std. Error	t-value	p-value
Intercept	16.6030	29.3813	0.565	0.581
synonymous.hom	-25.8861	58.6340	-0.441	0.666
phase: low	-0.6033	1.4133	-0.427	0.676
sex: male	0.5663	1.4056	0.403	0.693

Table S13: Results from two-sided generalized linear model (survival ~ synonymous.hom + phase + sex, family = binomial, data = mutations_fitness) where bold values indicate significant results. Asterisks show level of significance as P<0.001 ***, P<0.01 ** and P<0.05 *.

**Coefficents**	Estimate	Std. Error	z-value	p-value
Intercept	51.3237	21.2819	2.412	**0.0159***
synonymous.hom	-99.6908	41.3663	-2.410	**0.0160***
phase: low	-0.7841	0.9554	-0.821	0.4118
sex: male	-0.7572	0.9046	-0.837	0.4025

Table S14: Results from two-sided linear model (square.root.LRS ~ heterozygosity.kb + phase + sex, data = mutations_fitness).

**Coefficents**	Estimate	Std. Error	t-value	p-value
Intercept	2.0265	4.9901	0.406	0.691
heterozygosity.kb	0.1352	2.9370	0.046	0.964
phase: low	-0.4592	0.7339	-0.626	0.542
sex: male	0.4478	0.7502	0.597	0.560

Table S15: Results from two-sided linear model (log.age ~ heterozygosity.kb + phase + sex, data = mutations_fitness).

**Coefficents**	Estimate	Std. Error	t-value	p-value
Intercept	-0.13376	2.14476	-0.062	0.951
heterozygosity.kb	0.69101	1.26454	0.546	0.592
phase: low	0.01029	0.29275	0.035	0.972
sex: male	-0.06464	0.30944	-0.209	0.837

Table S16: Results from two-sided linear model (litter.size ~ heterozygosity.kb + phase + sex, data = mutations_fitness).

**Coefficents**	Estimate	Std. Error	t-value	p-value
Intercept	5.0095	9.5736	0.523	0.609
heterozygosity.kb	-0.8110	5.6346	-0.144	0.888
phase: low	-0.5268	1.4080	-0.374	0.714
sex: male	0.7621	1.4393	0.529	0.605

Table S17: Results from two-sided generalized linear model (survival ~ heterozygosity.kb + phase + sex, family = binomial, data = mutations_fitness) where bold values indicate significant results. Asterisks show level of significance as P<0.001 ***, P<0.01 ** and P<0.05 *.

**Coefficents**	Estimate	Std. Error	z-value	p-value
Intercept	-15.2858	6.0103	-2.543	**0.0110***
heterozygosity.kb	10.0137	3.8916	-2.411	**0.0159***
phase: low	-0.3757	0.8904	-0.422	0.6730
sex: male	-0.9517	0.9676	-0.984	0.3253

Table S18: Gene ontology of loss of function (LoF) variants with increased frequency in immigrant descendants compared with natives, individuals producing small litters (0 - 2 cubs) compared with large litters (6.67 – 9.5 cubs) and individuals with short lives (1 – 3 years) compared with long lives (4 – 9 years) respectively. Functions were based on literature search on mammalian studies.

LoF association	Scaffold	Position	Gene	Protein	Reference	Species	Biological process involved in:
**Ancestry**	NW_020356435.1	18481666	GRAMD2A	GRAM domain-containing protein 2A	Besprozvannaya et al. 2018	*Homo sapiens*	Calcium homeostasis
	NW_020356435.1	29177668	HTR7	5-hydroxotryptamine receptor 7	Romero-Reyes et al. 2021	*Mammalia*	Serotonin receptor
	NW_020356440.1	27095070	SEMA3G	Semaphorin 3G	Liu 2020; Oleari et al. 2021	*Mus musculus*	Adipogenesis and lipogenesis
	NW_020356446.1	27031833	NAAA	N-acylethanolamine acid amidase	Gorelik et al. 2018	*Rodentia*	Immunoregulatory enzyme
	NW_020356474.1	5043351	GMPPA	NTP_transferase domain-containing protein	Koehler et al. 2013; Franzka et al. 2021	*Homo sapiens, Mus musculus*	Mannose metabolism. Mutations can cause neuromuscular disorders
	NW_020356440.1	31390986	KIF9	Kinesin-like protein KIF9	Miyata et al. 2020; Chen et al. 2020	*Mus musculus*	Microtubule-based movement. Mutations can cause impaired sperm motility and male sterility.
	NW_020356575.1	154827	CCDC93	Coiled-coil domain-containing protein 93	Rimbert et al. 2020	*Homo sapiens, Mus musculus*	Protein recycling. Influences cholesterol levels
	NW_020356568.1	600355	RCBTB1	BTB domain-containing protein 1	Coppieters et al. 2016; Huhtaniemi et al. 2018	*Homo sapiens*	Chromosome condensation. Mutations can cause retinal disorders as well as ovarian insufficiency
	NW_020356633.1	223597	FGL1	Fibrinogen C-terminal domain-containing protein 1	Wang et al. 2019	*Mus musculus*	Immune inhibitory ligand that mediates T-cell suppression
	NW_020356440.1	27288956	PPM1M	Protein phosphatase 1M	Kamada et al. 2020	*Mammalia*	Phosphatase regulation
	NW_020356519.1	4709708	FBXO7	F-box protein 7	Ding et al. 2012; Ballesteros Reviriego et al. 2019	*Homo sapiens, Mus musculus*	Cell cycle and proteasome regulator. Mutations can affect T-cell survival, erythrocyte development and cause male sterility
	NW_020356466.1	17693141	TBCD	Tubulin-specific chaperone D	López Fanarraga et al. 2010	*Homo sapiens*	Centriologenesis and spindle organization
	NW_020356686.1	1190476	RHPN1	Rhophilin Rho GTPase binding protein 1	Lal et al. 2015	*Mus musculus*	Actin cytoskeleton organization
	NW_020356436.1	38717152	LOC112921411	—	—	—	—
	NW_020356489.1	12199540	LOC112925210	—	—	—	—
	NW_020356470.1	9777568	LOC112921721	—	—	—	—
**Longevity**	NW_020356437.1	6670264	ZNF503	Zinc finger protein 503	Shahi et al. 2015	*Homo sapiens*	Hindbrain and limb development
	NW_020356440.1	30964562	SMARCC1	SWI/SNF related, matrix associated actin dependent regulator of chromatin subfamily c member 1	Schaniel et al. 2009	*Mus musculus*	Self-renewal of stem cells and chromatin structuring
	NW_020356486.1	5233513	CDT1	DNA replication factor CDT1	Coulombe et al. 2013	*Mus musculus*	DNA replication licensing
	NW_020356539.1	6669648	ZNF217	Zinc finger protein 217	Zhai et al. 2019	*Rattus norvegicus*	Transcription regulator. Mutations can cause ovarian disorders
	NW_020356583.1	2278514	GAS2	Growth arrest specific 2-like protein 1	York et al. 2016; Chen et al. 2021	*Homo sapiens, Mus musculus*	Cytoskeletal regulation. Mutations can cause hearing loss and female reproductive deficiencies
	NW_020356634.1	2029296	CRLF2	Cytokine receptor-like factor 2	Lundström et al. 2012	*Homo sapiens*	Maturation of T-cells
	NW_020356624.1	2632813	ABHD1	AB hydrolase-1 domain-containing protein	Lord et al. 2013; Linke et al. 2020	*Mammalia*	Fatty acid biosynthesis and lipid metabolism
	NW_020356593.1	3806970	SETD1B	Histone-lysine N-methyltransferase SETD1B	Brici et al. 2017; Schmidt et al. 2018	*Mus musculus*	Histone modifying enzyme. Essential in haematopoesis and regulates oocyte expression. Mutations can cause female sterility as well as thrombycyte and lymphocyte deficiency.
	NW_020356569.1	5176098	MGAT3	Mannosyl-glycoprotein beta-1,4-N-acetylglucosaminyltransferase	Brandt et al. 2016	*Homo sapiens*	Stimulates lipid droplet growth
	NW_020356529.1	5684054	PATL1	PAT1 domain-containing protein	Scheller et al. 2007	*Homo sapiens*	mRNA translation
	NW_020356444.1	14877483	RPGRIP1	X-linked retinitis pigmentosa GTPase regulator-interacting protein 1	Narfström et al. 2012; Miyadera et al. 2012	*Canis lupus familiaris*	Retinal development. Mutations can cause retinal degenerative disorders
	NW_020356440.1	36505448	LOC112913139	—	—	—	—
	NW_020356473.1	724057	LOC112922177	—	—	—	—
	NW_020356489.1	12181586	LOC112925210	—	—	—	—
	NW_020356568.1	285211	LOC112933925	—	—	—	—
**Litter size**	NW_020356523.1	8436686	HSPG2	Heparan sulfate proteoglycan 2	Nicole et al. 2000; Lowe et al. 2014	*Homo sapiens, Mus musculus*	Cartilage formation. Mutations can cause various sceletal abnormalities
	NW_020356542.1	3393222	OSR1	Odd-skipped related transcription factor 1	Gao et al. 2009; Xie et al. 2013	*Mus musculus*	Embryonic cardiovascular and urogenital development
	NW_020356665.1	801554	ACACB	Acetyl-CoA carboxylase	Dong et al. 2021	*Homo sapiens*	Fatty acid synthesis and metabolism
	NW_020356472.1	15390696	ERMP1	Peptidase_M28 domain-containing protein 1	Garcia-Rudaz et al. 2007	*Rattus norvegicus*	Ovarian histogenesis. Mutations can cause polyovular follicles
	NW_020356529.1	3911568	RCN1	Reticulocalbin 1	Fukuda et al. 2007; Ding et al. 2015	*Homo sapiens*	Calcium ion binding. Stimulates microglial phagocytosis
	NW_020356632.1	1110603	LUZP6	Leucine zipper protein 6	Miller 2009	*Mammalia*	Transcriptional regulator
	NW_020356650.1	274975	CUNH22orf24	—	—	—	—
	NW_020356440.1	36505448	LOC112913139	—	—	—	—

Table S19: Results from two-sided linear mixed effect model (square.root.LRS ~ ancestry + phase + sex + (1 | den), data = ancestry_fitness) where bold values indicate significant results. Asterisks show level of significance as P<0.001 ***, P<0.01 ** and P<0.05 *.

**Fixed effects**	Estimate	Std. Error	t-value	p-value
Intercept	3.6099	0.4703	7.676	**1.64e-14*** **
ancestry: F2+F3	-1.0797	0.4344	-2.486	**0.01293***
ancestry: native	-0.8041	0.4378	-1.837	0.06625
phase: low	-0.7831	0.2924	-2.678	**0.00741****
sex: male	-0.1296	0.2906	-0.446	0.65559
**Random effects**	Variance	Std. Dev		
den	0.4024	0.6344		
Residual	1.3015	1.1408		

Table S20: Results from two-sided linear mixed effect model (log.age ~ ancestry + phase + sex + (1 | den), data = ancestry_fitness) where bold values indicate significant results. Asterisks show level of significance as P<0.001 ***, P<0.01 ** and P<0.05 *.

**Fixed effects**	Estimate	Std. Error	t-value	p-value
Intercept	1.45723	0.24762	5.885	**3.97e-09*** **
ancestry: F2+F3	-0.46277	0.23520	-1.968	**0.04912***
ancestry: native	-0.26866	0.24540	-1.095	0.27361
phase: low	-0.21663	0.17751	-1.220	0.22234
sex: male	0.02811	0.17424	0.161	0.87185
**Random effects**	Variance	Std. Dev		
den	0.02985	0.1728		
Residual	0.54947	0.7413		

Table S21: Results from two-sided linear mixed effect model (litter.size ~ pair + phase + (1 | den), data = ancestry_fitness) where bold values indicate significant results. Asterisks show level of significance as P<0.001 ***, P<0.01 ** and P<0.05 *.

**Fixed effects**	Estimate	Std. Error	t-value	p-value
Intercept	9.947	1.024	9.718	**0.00e+00*** **
pair: F1-4XF1-4	-4.226	1.128	-3.745	**0.00018*** **
pair: nativeXF1-4	-5.410	1.015	-5.330	**9.82e-08*** **
pair: nativeXnative	-4.154	1.044	-3.981	**6.85e-05*** **
phase: low	1.454	0.445	3.269	**0.00108****
**Random effects**	Variance	Std. Dev		
den	0.2329	0.4826		
Residual	5.2017	2.2807		

References for table S18

1. Ballesteros Reviriego et al. (2019) FBXO7 sensitivity of phenotypic traits elucidated by a hypomorphic allele. *PloS one* 14, e0212481

2. Besprozvannaya M, Dickson E, Li H, Ginburg KS, Bers DM, Auwerx J & Nunnari J (2018) GRAM domain proteins specialize functionally distinct ER-PM contact sites in human cells. *eLife* 7, e31019

3. Brandt C, McFie PJ & Stone SJ (2016) Biochemical characterization of human acyl coenzyme A: 2-monoacylglycerol acyltransferase-3 (MGAT3). *Biochemical & Biophysical Research Communications* 475, 264-270

4. Brici D et al. (2017) Setd1b, encoding a histone 3 lysine 4 methyltransferase, is a maternal effet gene required for the oogenic gene expression program. *Development* 144, 2606-2617

5. Chen K, Cho SY, Zhang Y, Beck A & Segall JE (2020) A role for KIF9 in male fertility. *bioRxiv*

6. Chen T et al. (2021) Cochlear supporting cells require GAS2 for cytoskeletal architecture and hearing. *Developmental Cell* 56, 1526-1540

7. Coppieters F et al. (2016) Isolated and syndromic retinal dystrophy caused by biallelic mutations in RCBTB1, a gene implicated in ubiquitination. *The American Journal of Human Genetics* 99, 470-480

8. Coulombe P, Grégoire D, Tsanov N & Méchali M (2013) A spontaneous Cdt1 mutation in 129 mouse strains reveals a regulatory domain restraining replication licensing. *Nature Communications* 4, 2065

9. Ding K et al. (2012) Genetic loci implicated in erythroid differentiation and cell cycle regulation are associated with red blood cell traits. *Mayo Clinic Proceedings* 87, 461-474

10. Ding Y, Caberoy NB, Guo F, LeBlanc ME, Zhang C, Wang W, Wang F, Chen R & Li W (2015) Reticulocalbin-1 facilitates microglial phagocytosis. *PloS one* 10, e0126993

11. Dong Z, Lei X, Kujawa SA, Bolu N, Zhao H & Wang C (2021) Identification of core in obese type 2 diabetes patients using bioinformatics analysis. *Adipocyte* 10, 310-321

12. Franzka P et al. (2021) GMPPA defects cause a neuromuscular disorder with α-dystroglycan hyperglycosylation. *The Journal of Clincical Investigation* 131, e139076

13. Fukuda T et al. (2007) Distribution and variable expression of secretory pathway protein reticulocalbin in normal human organs and non-neoplastic pathological conditions. *Journal of Histochemistry & Cytochemistry* 55, 335-345

14. Garcia-Rudaz C, Luna F, Tapia V, Kerr B, Colgin L, Galimi F, Dissen GA, Rawlings ND & Ojeda SR (2007) Fxna, a novel gene differentially expressed in the rat ovary at the time of folliculogenesis, is required for normal ovarian histogenesis. *Development* 134, 945-957

15. Gao Y, Lan Y, Ovitt CE & Jiang R (2009) Functional equivalence of the zinc finger transcription factors Osr1 and Osr2 in mouse development. *Developmental Biology* 328, 200-209

16. Gorelik A, Gebai A, Illes K, Piomelli D & Nagar B (2018) Molecular mechanism of activation of the immunoregulatory amidase NAAA. *Proceedings of the National Academy of Sciences* 115, E10032-E10040

17. Huhtaniemi I et al. (2018) Advances in the molecular pathophysiology, genetics and treatment of primary ovarian insufficiency. *Trends in Endocrinology & Metabolism* 29, 400-419

18. Kamada R, Kudoh F, Ito S, Tani I, Janairo JIB, Omichinski JG & Sakaguchi K (2020) Metal-dependent Ser/Thr protein phosphatase PPM family: evolution, structures, diseases and inhibitors. *Pharmacology & Therapeutics* 215, 107622

19. Koehler K et al. (2013) Mutations in GMPPA cause a glycosylation disorder characterized by intellectual disability and autonomic dysfunction. *American Journal of Human Genetics* 93, 727-734

20. Lal MA, Andersson A-C, Katayama K, Xiao Z, Nukui M, Hultenby K, Wernerson A & Tryggvason K (2015) Rhophilin-1 is a key regulator of the podocyte cytoskeleton and is essential for glomerular filtration. *Journal of the American Society of Nephrology* 26, 647-662

21. Linke V et al. (2020) A large-scale genome-lipid association map guides lipid identification. *Nature Metabolism* 2, 1149-1162

22. Liu M, Xie S, Liu W, Li J, Li C, Huang W, Li H, Song J & Zhang H (2020) Mechanism of SEMA3G knockdown-mediated attenuation of high-fat diet-induced obesity. *Journal of Endocrinology* 244, 223-236

23. López Fanarraga M, Bellido J, Jaén C, Villegas JC & Zabala JC (2010) TBCD links centriologenesis, spindle microtubule dynamics, and midbody abscission in human cells. *PloS one* 5, e8846

24. Lord CC, Thomas G & Brown JM (2013) Mammalian alpha beta hydrolase domain (ABHD) proteins: lipid metabolizing enzymes at the interface of cell signaling and energy metabolism. *Biochimica et Biophysica Acta (BBA) – Molecular and Cell Biology of Lipids* 1831, 792-802

25. Lowe DA, Lepori-Bui N, Fomin PV, Sloofman LG, Zhou X, Farach-Carson MC, Wang L & Kirn-Safran CB (2014) *Calcified Tissue International* 95, 29-38

26. Lundström W, Fewkes NM & Mackall CL (2012) IL-7 in human health and disease. *Seminars in Immunology* 24, 218-224

27. Miller M (2009) The importance of being flexible: the case of basic region leucine zipper transcriptional regulators. *Current Protein & Peptide Science* 10, 244-269

28. Miyadera K, Kato K, Boursnell M, Mellersh CS & Sargan DR (2012) Genome-wide association study in RPGRIP1-/- dogs identifies a modifier locus that determines the onset of retinal degeneration. *Mammalian Genome* 23, 212-223

29. Miyata H, Shimada K, Morohoshi A, Oura S, Matsumura T, Xu Z, Oyama Y & Ikawa M (2020) Testis-enriched kinesin KIF9 is important for progressive motility in mouse spermatozoa. *The Journal of the Federation of American Societies for Experimental Biology* 34, 5389-5400

30. Narfström K, Jeong M, Hyman J, Madsen RW & Begström TF (2012) Assessment of hereditary retinal degeneration in the English springer spaniel dog and disease relationship to an RPGRIP1 mutation. *Stem Cells International* 2012

31. Nicole S et al. (2000) Perlecan, the major proteoglycan of basement membranes, is altered in patients with Schwartz-Jampel syndrome (chondrodystrophic myotonia). *Nature Genetics* 26, 480-483

32. Oleari R et al. (2021) A novel SEMA3G mutation in two siblings affected by syndromic GnRH deficiency. *Neuroendocrinology* 111, 421-441

33. Rimbert A et al. (2020) A common variant in CCDC93 protects against myocardial infarction and cardiovascular mortality by regulating endosomal trafficking of low-density lipoprotein receptor. *European Heart Journal* 41, 1040-1053

34. Romero-Reyes J, Molina-Hernández A, Díaz NF, Camacho-Arroyo I (2021) Role of serotonin in vertebrate embryo development. *Reproductive Biology* 21, 100475

35. Schaniel C, Ang Y-A, Ratnakumar K, Cormier C, James T, Bernstein E, Lemischka IR & Paddison PJ (2009) Smarcc1/Baf155 couples self-renewal gene repression with changes in chromatin structure in mouse embryonic stem cells. *Stem Cells* 27, 2979-2991

36. Scheller N, Resa-Infante P, de la Luna S, Galao RP, Albrecht M, Kaestner L, Lipp P, Lengauer T, Meyerhans A, Díez J (2007) Identification of PatL1, a human homolog to yeast P body component Pat1. *Biochimica et Biophysica Acta – Molecular Cell Research* 1773, 1786-1792

37. Schmidt K et al. (2018) The H3K4 methyltransferase Setd1b is essential for hematopoietic stem and progenitor cell homeostasis in mice. *eLife* 7, e27157

38. Shahi P, Slorach EM, Wang CY, Chou J, Lu A, Ruderisch A & Werb Z (2015) The transcriptional repressor ZNF503/Zeppo2 promotes mammary epithelial cell proliferation and inhances cell invasion. *Journal of Biological Chemistry* 290, 3803-3813

39. Shaniel C, Ang Y-S, Ratnakumar K, Cormier C, James T, Bernstein E, Lemischka IR & Paddison PJ (2009) Smarcc1/Baf155 couples self-renewal gene repression with changes in chromatin structure in mouse embryonic stem cells. *Stem Cells* 27, 2979-2991

40. Wang J et al. (2019) Fibrinogen-like protein 1 is a major immune inhibitory ligand of LAG-3. *Cell* 176, 334-347

41. Xie J, Yoon J, Yang SS, Lin SH & Huang CL (2013) WNK1 protein kinase regulates embryonic cardiovascular development through the OSR1 signaling cascade. *Journal of Biological Chemistry* 288, 8566-8574

42. York JP, Ren YA, Zeng J, Zhang B, Wang F, Chen R, Liu J, Xia X & Zhang P (2016) Growth arrest specific 2 (GAS2) is a critical mediator of germ cell cyst breakdown and folliculogenesis in mice. *Scientific Reports* 6, 34956

43. Zhai J, Li S, Cheng X, Chen ZJ, Li W & Du Y (2020) A candidate pathogenic gene, zinc finger 217 (ZNF217), may contribute to polycystic ovary syndrome through prostaglandin E2. *Acta Obstetricia et Gynecologica Scandinavica* 99, 119-126
