# Supplementary material for: Strongly deleterious mutations influence reproductive output and longevity in an endangered population
Source: Nat Commun. 2024 Sep 27;15:8378. doi: 10.1038/s41467-024-52741-4 (PMC11436772; doi:10.1038/s41467-024-52741-4)
Supplement: Supplementary file 3 — Reporting Summary [file 41467_2024_52741_MOESM3_ESM.pdf]

nature portfolio

Corresponding author(s): Malin Hasselgren

Last updated by author(s): Sep 6, 2024

Reporting Summary

Nature Portfolio wishes to improve the reproducibility of the work that we publish. This form provides structure for consistency and transparency in reporting. For further information on Nature Portfolio policies, see our Editorial Policies and the Editorial Policy Checklist.

Statistics

For all statistical analyses, confirm that the following items are present in the figure legend, table legend, main text, or Methods section.

n/a	Confirmed	
☐	☒	The exact sample size (*n*) for each experimental group/condition, given as a discrete number and unit of measurement
☐	☒	A statement on whether measurements were taken from distinct samples or whether the same sample was measured repeatedly
☐	☒	The statistical test(s) used AND whether they are one- or two-sided<br>*Only common tests should be described solely by name; describe more complex techniques in the Methods section.*
☐	☒	A description of all covariates tested
☐	☒	A description of any assumptions or corrections, such as tests of normality and adjustment for multiple comparisons
☐	☒	A full description of the statistical parameters including central tendency (e.g. means) or other basic estimates (e.g. regression coefficient) AND variation (e.g. standard deviation) or associated estimates of uncertainty (e.g. confidence intervals)
☐	☒	For null hypothesis testing, the test statistic (e.g. *F*, *t*, *r*) with confidence intervals, effect sizes, degrees of freedom and *P* value noted<br>*Give P values as exact values whenever suitable.*
☒	☐	For Bayesian analysis, information on the choice of priors and Markov chain Monte Carlo settings
☒	☐	For hierarchical and complex designs, identification of the appropriate level for tests and full reporting of outcomes
☐	☒	Estimates of effect sizes (e.g. Cohen's *d*, Pearson's *r*), indicating how they were calculated

Our web collection on statistics for biologists contains articles on many of the points above.

Software and code

Policy information about availability of computer code

Data collection	No software was used

Data analysis

Genomic sequences were analyzed through a bioinformatic pipeline on genome erosion and statistical tests were carried out in R v.3.6.1. First, adapters were trimmed from raw reads using Trimmomatic version 0.36. Trimmed reads were mapped to a red fox de novo assembly using the Burrows-Wheeler Aligner (BWA) version 0.7.17. BAM-files were indexed and sorted using Samtools version 1.8, duplicates were removed using Picard version 2.10.3 and indels were realigned using GATK version 3.7. We used Qualimap version 2.2 for quality control and to obtain mean depth of coverage. Variant calling was performed using bcftools mpileup version 1.8. We used the same software to filter out sites with a quality score lower than 30 and SNPs within 5 bp of indels. We filtered out hard masked repeat regions using BEDTools version 2.27.1. We annotated synonymous and non-synonymous variants within and in proximity of coding regions by using the red fox annotation. We used SNPeff version 4.3 to generate a database for red fox, using the protein sequences extracted from its annotation. We removed in-frame stop codons from the annotation using the -V option in cufflinks version 2.2.1.

All following tests were performed in R v.4.4.0. We performed non-parametric Mann-Whitney U-tests to compare the proportion of deleterious variants in the re-sequenced native foxes versus those with immigrant ancestry. We performed linear models to estimate the effect of mutational load and genome-wide heterozygosity on average litter size, longevity and lifetime reproductive success in the re-sequenced foxes that survived to adulthood, controlling for sex and phase of the rodent cycle. We performed generalized linear models with binomial distribution to estimate the effect of mutational load and genome-wide heterozygosity on juvenile survival, also controllig for sex and phase of the rodent cycle. To test if there was an effect of individual ancestry on litter size, longevity or lifetime reproductive success, we performed three linear mixed effect models using phase of the rodent cycle as fixed effect and natal den as random effect. For the longevity and LRS analyses, the sex of each fox was also included as a fixed effect.

For manuscripts utilizing custom algorithms or software that are central to the research but not yet described in published literature, software must be made available to editors and reviewers. We strongly encourage code deposition in a community repository (e.g. GitHub). See the Nature Portfolio guidelines for submitting code & software for further information.

Data

Policy information about availability of data

All manuscripts must include a data availability statement. This statement should provide the following information, where applicable:

- Accession codes, unique identifiers, or web links for publicly available datasets
- A description of any restrictions on data availability
- For clinical datasets or third party data, please ensure that the statement adheres to our policy

Data availability
The re-sequencing data generated in this study have been deposited in the European Nucleotide Archive (ENA) under the accession numbers: PRJEB76449 [https://www.ebi.ac.uk/ena/browser/view/PRJEB76449], PRJEB43377 [https://www.ebi.ac.uk/ena/browser/view/PRJEB43377] and PRJEB55788 [https://www.ebi.ac.uk/ena/browser/view/PRJEB55788]. The datasets generated in this study are available in the Dryad database under accession code: [https://doi.org/10.5061/dryad.7wm37pw2r]. Source data are provided with this paper.

Code availability
The r-code generated in this study are available in the Dryad database under accession code: [https://doi.org/10.5061/dryad.7wm37pw2r].

Research involving human participants, their data, or biological material

Policy information about studies with human participants or human data. See also policy information about sex, gender (identity/presentation), and sexual orientation and race, ethnicity and racism.

Reporting on sex and gender

Use the terms sex (biological attribute) and gender (shaped by social and cultural circumstances) carefully in order to avoid confusing both terms. Indicate if findings apply to only one sex or gender; describe whether sex and gender were considered in study design; whether sex and/or gender was determined based on self-reporting or assigned and methods used.
Provide in the source data disaggregated sex and gender data, where this information has been collected, and if consent has been obtained for sharing of individual-level data; provide overall numbers in this Reporting Summary. Please state if this information has not been collected.
Report sex- and gender-based analyses where performed, justify reasons for lack of sex- and gender-based analysis.

Reporting on race, ethnicity, or other socially relevant groupings

Please specify the socially constructed or socially relevant categorization variable(s) used in your manuscript and explain why they were used. Please note that such variables should not be used as proxies for other socially constructed/relevant variables (for example, race or ethnicity should not be used as a proxy for socioeconomic status).
Provide clear definitions of the relevant terms used, how they were provided (by the participants/respondents, the researchers, or third parties), and the method(s) used to classify people into the different categories (e.g. self-report, census or administrative data, social media data, etc.)
Please provide details about how you controlled for confounding variables in your analyses.

Population characteristics

Describe the covariate-relevant population characteristics of the human research participants (e.g. age, genotypic information, past and current diagnosis and treatment categories). If you filled out the behavioural & social sciences study design questions and have nothing to add here, write "See above."

Recruitment

Describe how participants were recruited. Outline any potential self-selection bias or other biases that may be present and how these are likely to impact results.

Ethics oversight

Identify the organization(s) that approved the study protocol.

Note that full information on the approval of the study protocol must also be provided in the manuscript.

Field-specific reporting

Please select the one below that is the best fit for your research. If you are not sure, read the appropriate sections before making your selection.

☐ Life sciences ☐ Behavioural & social sciences ☒ Ecological, evolutionary & environmental sciences

For a reference copy of the document with all sections, see nature.com/documents/nr-reporting-summary-flat.pdf

Ecological, evolutionary & environmental sciences study design

All studies must disclose on these points even when the disclosure is negative.

Study description	The study is carried out on a natural population of arctic foxes in Sweden. We investigated how putatively deleterious mutations and genome-wide heterozygosity influenced individual fitness, whether an immigration event altered the proportion of deleterious mutations in the population and whether individual ancestry influenced fitness. For fitness traits, lifetime reproductive success, longevity, litter size and first year survival were used. Individual ancestry were based on whether foxes were of native origin or whether they had immigrant ancestry. These were also divided into different generations (F1-F4). The mutations were divided into three categories: synonymous, missense and loss of function mutations. When we tested whether individual ancestry influenced fitness we included sex and phase of the rodent cycle as fixed effects and natal den as a random effect.
Research sample	The majority of the foxes used in this study were sampled from the same subpopulation, the southernmost subpopulation in Sweden, which is very small and isolated. We used 30 arctic foxes for whole genome sequencing. These were selected to represent all founder and immigrant lineages and to get a variation of different inbreeding levels (according to the previously constructed pedigree) and fitness levels (survival and reproduction). The samples spanned from 2001 (when the population was founded) to 2018. We also used a larger genealogical dataset based on the previously published pedigree to investigate whether individual ancestry influenced fitness. These included all litters with known ancestry (121 litters) produced from 2010 (when the first immigration occurred) up until 2019 (the final year of the constructed pedigree). When investigating whether individual ancestry influenced longevity and lifetime reproductive success we included all individuals with known ancestry born between 2010 and 2015 that survived to adulthood (85 arctic foxes). In addition, to increase sample size in the genomic dataset, 7 re-sequenced individuals from a more northwards located subpopulation were used. These individuals were sampled during 2005-2019. This subpopulation had also been subjected to immigration and the samples consisted of both natives and immigrant descendants. The two subpopulations are isolated from each other but share recent demographic histories.
Sampling strategy	Every summer, all known den sites are visited, and if litters are present cubs are ear tagged and genetically sampled. During the study period we managed to sample from 93% of all reproductive dens and these were included in the demographic dataset of the study. For the genomic dataset, the 37 re-sequenced genomes were the number of available re-sequenced genomes with corresponding fitness data.
Data collection	Data is collected during July every summer and is collected by our research group, rangers from the county board administrations and volunteering field workers. Fitness traits are documented by identifying individual foxes based on their unique ear tag combinations as well as from genetical samples during pedigree construction. Moreover, snap trapping of rodents is carried out yearly at a fixed number of dens to estimate the phase of the rodent cycle. This data was retrieved from previous studies.
Timing and spatial scale	Data was collected annually from 2001 to 2019.
Data exclusions	For all analyses on the effect of genomic variation on fitness, foxes with unknown year of birth were excluded (2) since we could not control for the phase of the rodent cycle for these individuals. When investigating whether the proportion of deleterious mutations influenced litter size, LRS and age, we excluded individuals that died before reaching adulthood (14 foxes).<br><br>When we used the larger demographic dataset to investigate whether individual ancestry influenced litter size we excluded litters with unknown ancestry (45 litters). When investigating whether ancestry influenced longevity and lifetime reproductive success we also excluded individuals born after 2015 since few individuals had been recorded to reach adulthood due to limited data available.
Reproducibility	No experiments were carried out in this study
Randomization	No experiments were carried out in this study
Blinding	Blinding was not possible due to the opportunistic nature of the sampling. However, during data collection, the ancestry of individual foxes is usually unknown.

Did the study involve field work? ☒ Yes ☐ No

Field work, collection and transport

Field conditions	The fieldwork is accomplished every summer during July in the Swedish mountain tundra. During this month all known arctic fox dens are visited, and number of litters are recorded and cubs are counted and ear tagged. Ear tagged foxes from previous years are identified to monitor individual fitness. All inhabited dens are observed for a minimum of 24 hours. Weather conditions vary throughout the fieldseason between sun, snow, rain and wind.

Location	The arctic fox population in Scandinavia is small and fragmented and resides in the mountain tundra. The subpopulation in this study is located in Helagsfjällen (3 400 km2), Jämtland county (62 °N, 12 °E) and is the most southern and geographically isolated subpopulation in Sweden.
Access & import/export	The research compiles with all relevant ethics regulations, including permits to work in protected habitats, approved by the county board administration of Jämtland (521-4797-2017, 521-2593-2017) and Västerbotten (521-3191-2014, 521-4640-2019). Permits to catch, handle and eartag foxes were approved within the ethical permit from the Swedish Board of Agriculture (A49-01, A36-11, A130-07, A18-14, A10-17, A130-07) and additional allowances from Swedish Environmental Protection Agency (412-4191-03 Nf, 412-5362-04 Nf, 412-7884-07, NV-01959-14, 412-35-99 Nf, NV-02547-17).
Disturbance	Distrubance includes camping near the arctic fox den sites as well as capture and handling of individual foxes. Distrubance is minimized by camping at a minimum of 200 metres from the den at a maximum of three nights. When foxes are captured stress of the animals is minimized by keeping them in a sack to keep them calm as well as making the procedure as quick as possible (usually less than 5 minutes of handling).

Reporting for specific materials, systems and methods

We require information from authors about some types of materials, experimental systems and methods used in many studies. Here, indicate whether each material, system or method listed is relevant to your study. If you are not sure if a list item applies to your research, read the appropriate section before selecting a response.

Materials & experimental systems

n/a	Involved in the study
☒	☐ Antibodies
☒	☐ Eukaryotic cell lines
☒	☐ Palaeontology and archaeology
☐	☒ Animals and other organisms
☒	☐ Clinical data
☒	☐ Dual use research of concern
☒	☐ Plants

Methods

n/a	Involved in the study
☒	☐ ChIP-seq
☒	☐ Flow cytometry
☒	☐ MRI-based neuroimaging

Animals and other research organisms

Policy information about studies involving animals; ARRIVE guidelines recommended for reporting animal research, and Sex and Gender in Research

Laboratory animals	The study did not use laboratory animals
Wild animals	This study includes complete genomes of 37 individual arctic foxes and fitness and genealogical data of 85 arctic foxes (traits: life span and lifetime reproductive success) and 121 litters (trait: litter size). The foxes have been observed and captured in their natural habitat, in the Swedish mountain tundra. Captured foxes are never transported from their natural habitat, but are handled at their respective den site. Foxes are generally caught as cubs (1-2 months old) at their natal den but a small number of individuals have been caught as adults (> 1 year old), either at their natal den or at their reproductive territory. The foxes are caught using tomahawk live traps baited with food. Caught individuals are weighted, measured, sex determined and ear tagged and subsequently released at their den site.
Reporting on sex	Individuals are sex determined in the field when they are handled, and sex is also confirmed later through genetic analyses. In this study, both females and males were used. For the whole genome data, the sex chromosomes were filtered out. Both males and females were included in the statistical analyses. For the fitness analyses, sex was controlled for as a fixed effect.
Field-collected samples	When arctic foxes are eartagged, we obtain a small piece of tissue that is used for DNA extractions. The tissue is kept in 99% ethanol and stored in -20 degrees celsius up until extraction.
Ethics oversight	The research compiles with all relevant ethics regulations, including permits to work in protected habitats, approved by the county board administration of Jämtland (521-4797-2017, 521-2593-2017) and Västerbotten (521-3191-2014, 521-4640-2019). Permits to catch, handle and eartag foxes were approved within the ethical permit from the Swedish Board of Agriculture (A49-01, A36-11, A130-07, A18-14, A10-17, A130-07) and additional allowances from Swedish Environmental Protection Agency (412-4191-03 Nf, 412-5362-04 Nf, 412-7884-07, NV-01959-14, 412-35-99 Nf, NV-02547-17).

Note that full information on the approval of the study protocol must also be provided in the manuscript.

Plants

Seed stocks	No plants used
Novel plant genotypes	No plants used
Authentication	No plants used
